# Aging epicardial adipose tissue: a metabolic-endocrine network driving vascular calcification

**DOI:** 10.3389/fendo.2026.1817572

**Published:** 2026-04-17

**Authors:** Lizhen Zhou, Jishen Li, Ziyang Dong, Xiaxia Du, Yuqi Sun, Shan Tong

**Affiliations:** 1 School of Clinical Medicine, Hainan Medical University, Haikou, China; 2 General Practice Department, Hainan General Hospital, Haikou, China; 3 Hainan Affiliated Hospital of Hainan Medical University, Haikou, China

**Keywords:** age-related metabolic dysfunction, coronary artery calcification, epicardial adipose tissue, inflammation, metabolic-endocrine network, microRNA

## Abstract

Coronary artery calcification (CAC) is a hallmark of vascular aging and a major contributor to cardiovascular morbidity in the elderly. Recent evidence has identified epicardial adipose tissue (EAT) as a metabolically active endocrine organ whose age-related dysfunction critically contributes to this process. During aging, EAT undergoes a profound phenotypic switch—from a protective metabolic reservoir to a pathogenic secretory neighbor—that actively drives CAC progression. This review synthesizes current evidence to propose a novel conceptual framework: aged EAT orchestrates a multi-tiered and interactive metabolic-endocrine network that accelerates vascular calcification. At the core of this network lies a mutually reinforcing axis of chronic inflammation and oxidative stress, both fueled by underlying metabolic dysregulation. Built upon this foundation, dysregulated autophagy and apoptosis govern cellular fate decisions, while pathological vascular remodeling reshapes the extracellular matrix. Superimposed on these layers, a spectrum of dysregulated microRNAs acts as a master regulatory tier, integrating metabolic, inflammatory, and oxidative signals to amplify the entire network. By deciphering the complex crosstalk within this system, we identify key nodes where metabolic and endocrine signals converge—positioning the aged EAT as both a sensor and driver of vascular pathology. We conclude that targeting this metabolic-endocrine network offers a promising strategic avenue for mitigating age-related CAC, opening new frontiers for therapeutic intervention.

## 1 Introduction

Coronary artery calcification (CAC), a late-stage manifestation of atherosclerosis, progressively intensifies with age and serves as a major predictor of cardiovascular events and mortality in the elderly (1, 2). Despite its significant clinical implications, therapeutic options for established CAC remain strikingly limited. This reality underscores an urgent need to elucidate its underlying mechanisms—a crucial step for developing novel preventive and therapeutic strategies (3).

In recent years, epicardial adipose tissue (EAT)—a metabolically active endocrine organ situated in direct anatomical proximity to the coronary arteries—has emerged as a key player in cardiovascular disease (4, 5). Beyond its role as a passive fat depot, EAT actively secretes a wide array of adipokines and signaling molecules that influence myocardial and vascular function. Both basic and clinical studies indicate that aging not only increases EAT volume but also drives profound metabolic and endocrine dysfunction. This pathological remodeling transforms EAT from a cardioprotective reservoir into a dysfunctional source that releases a dysregulated array of pro-inflammatory and pro-calcific mediators (6, 7). Consequently, EAT is now regarded as a critical interface linking systemic aging with local coronary pathology.

However, how these scattered pieces of evidence across multiple pathways interact to form a coherent pathogenic network—and whether there exist dominant pathways or targetable nodes within it—remains to be systematically elucidated.

To address this gap, this review proposes that age-related metabolic-endocrine dysfunction of EAT serves as the central hub driving a complex, multi-tiered network that culminates in coronary artery calcification. First, we will delineate the pathological transition of EAT from a protective “guardian” to a harmful “disruptor” during aging, with a focus on its underlying metabolic alterations. Subsequently, following a hierarchical analytical framework, we will reveal how chronic inflammation and oxidative stress—both fueled by metabolic dysregulation—constitute a mutually reinforcing core axis. Building upon this axis, we will dissect how imbalanced autophagy and apoptosis regulate cellular fate decisions, and how pathological vascular remodeling restructures the extracellular matrix. Finally, we will elucidate how a spectrum of dysregulated microRNAs acts as an upstream regulatory layer, integrating metabolic, inflammatory, and oxidative signals to orchestrate the entire network. The review will conclude by discussing the implications of this metabolic-endocrine network model for understanding disease mechanisms and developing targeted, metabolism-focused therapies.

## 2 Aging promotes coronary artery calcification

CAC is a well-established hallmark of advanced atherosclerosis, and its severity is strongly associated with an increased risk of myocardial infarction and cardiac death (2). Among numerous risk factors, aging stands out as one of the strongest and most independent clinical predictors of CAC (8, 9). Epidemiological data demonstrate that both the prevalence and severity of CAC rise markedly with age. Clinically, elderly patients with CAC often present a therapeutic challenge, facing limited treatment options and a poorer prognosis (8).

## 3 Age-related pathological remodeling of EAT

Epicardial adipose tissue (EAT) is a unique visceral fat depot situated on the surface of the heart, sharing the microcirculation with the underlying myocardium and lacking a fascial barrier (10, 11). This anatomical intimacy positions EAT as a direct modulator of cardiac and coronary function. Beyond its structural role, EAT functions as a metabolically active endocrine organ, secreting a diverse repertoire of adipokines, cytokines, and signaling molecules that influence local vascular homeostasis (5, 7). Under physiological conditions, EAT supports cardiac energetics by storing and releasing fatty acids as needed, while simultaneously maintaining a protective secretory profile characterized by anti-inflammatory adipokines such as adiponectin (12). This delicate metabolic and endocrine balance enables EAT to act as a beneficial metabolic reservoir and a guardian of coronary health.

### 3.1 Age-related alterations: from quantitative to qualitative shifts

Aging profoundly alters EAT at both the structural and functional levels. First, its volume increases substantially. Consistent clinical imaging studies confirm that both EAT thickness and total volume rise with age—an association that may even surpass conventional metrics like body mass index (13).

More critically, parallel to this volumetric expansion, the metabolic and endocrine phenotype of EAT undergoes a fundamental deterioration (14). The inherent thermogenic capacity of EAT, conferred by uncoupling protein 1 (UCP-1) expression, declines with age, reflecting impaired mitochondrial function and reduced energy dissipation (15, 16). This metabolic deficit is accompanied by a profound reversal of its secretory profile: expression of pro-inflammatory factors (e.g., IL-6, TNF-α) is upregulated, while secretion of protective, metabolically beneficial adipokines (e.g., adiponectin) is markedly reduced (17). At the microscopic level, this functional decline is further compounded by the infiltration and polarization of pro-inflammatory M1 macrophages, which replace the resident anti-inflammatory M2 population and amplify local metabolic-inflammatory crosstalk (7, 18).

### 3.2 The pathological core: chronic inflammation and metabolic dysregulation

In summary, the core hallmark of age-related EAT dysfunction is a symbiotic state of chronic low-grade inflammation and local metabolic imbalance (19). This pathological synergy arises from the inherent metabolic-endocrine nature of EAT itself: its adipocytes and resident immune cells become locked in a self-reinforcing crosstalk, characterized by excessive secretion of pro-inflammatory adipokines, chemokines, and reactive oxygen species (6, 7). The metabolic derangement—including impaired mitochondrial function, altered lipid handling, and reduced adiponectin production—fuels this inflammatory milieu, while the ensuing inflammation further disrupts local metabolic homeostasis, creating a vicious metabolic-inflammatory cycle (20).

Due to EAT’s direct anatomical proximity to the coronary arteries, these pathological mediators act directly and continuously on the vascular wall (21). This sustained assault initiates and accelerates endothelial injury, vascular smooth muscle cell (VSMC) transdifferentiation, and the progression of atherosclerosis (6, 22, 23). Consequently, EAT undergoes a complete functional transformation—from a metabolically protective endocrine depot to a pathogenic metabolic hub that actively promotes vascular disease.

## 4 The aged EAT as a central hub in the metabolic-endocrine network driving CAC

**Figure 1:**
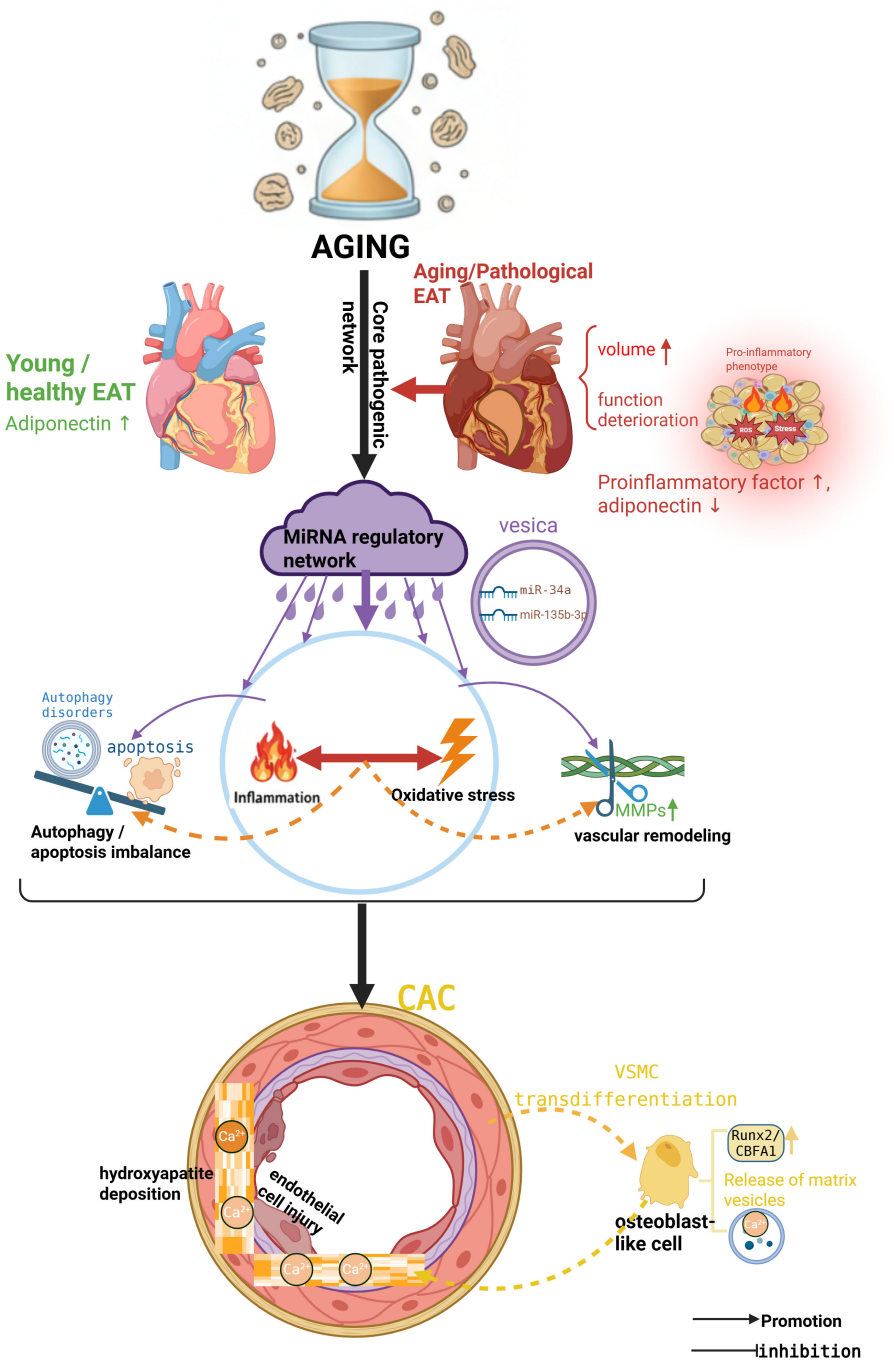
A multi-tiered and interactive network orchestrated by aged epicardial adipose tissue drives coronary artery calcification.This conceptual overview illustrates the central paradigm of this review. Pathological remodeling of epicardial adipose tissue (EAT) with aging transitions this tissue from a protective guardian to a pathogenic neighbor. The aged EAT acts as a central conductor, orchestrating a synergistic network of five interconnected pathological tiers to promote coronary artery calcification (CAC): chronic inflammation, oxidative stress, dysregulation of cellular fate (autophagy/apoptosis), vascular remodeling, and an overarching dysregulated microRNA (miRNA) network. Solid arrows indicate the primary initiating influence of aged EAT on each tier and the convergence on CAC. Dashed lines between modules emphasize the critical crosstalk and feedback loops that amplify the overall pro-calcific signal. The specific mechanisms of each tier are dissected in the subsequent figures. (Created with BioRender.com).

Driven by the age-related metabolic and endocrine alterations detailed above, the dysfunctional EAT transforms into a pathological secretory hub, continuously releasing a cascade of mediators into the adjacent coronary microenvironment. These mediators—ranging from pro-inflammatory adipokines to reactive oxygen species and dysregulated microRNAs—do not act in isolation. Rather, they coalesce into a hierarchical and interactive metabolic-endocrine network that systematically drives coronary artery calcification (**Figure 1**).

Within this network, a self-reinforcing core axis emerges, wherein chronic inflammation and oxidative stress—both fueled by underlying metabolic dysregulation—sustain and amplify each other. Built upon this foundation, two interconnected pathological layers execute the calcification program: first, the imbalance between autophagy and apoptosis governs the fate of vascular cells, determining their survival or death; second, pathological vascular remodeling degrades and reorganizes the extracellular matrix, transforming it from a structural scaffold into a pro-calcific “landing field.” Superimposed upon and integrating these layers, a dysregulated miRNA network acts as an upstream regulatory hub, precisely coordinating metabolic, inflammatory, and oxidative signals to amplify the entire pathological cascade.

The following sections will dissect this complex metabolic-endocrine network layer by layer, beginning with the most immediate and potent arm of the assault: inflammation.

### 4.1 Inflammation

**Figure 2:**
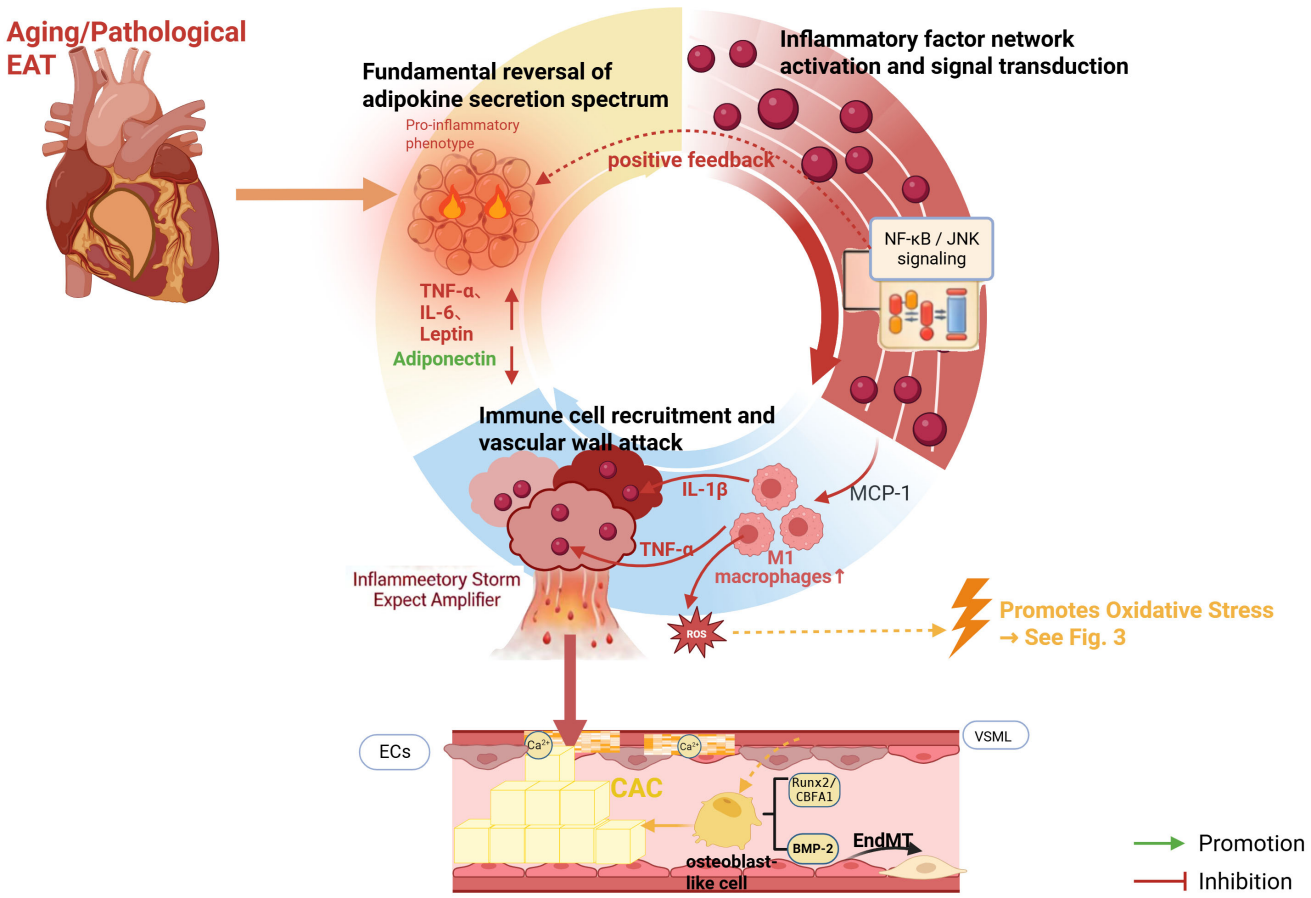
The inflammatory cascade: a three-tiered attack from aged epicardial adipose tissue on the coronary artery.This schematic delineates the stepwise inflammatory cascade initiated by aged EAT. The process begins with a fundamental shift in the adipokine secretome (Tier 1), characterized by an increase in pro-inflammatory mediators (e.g., tumor necrosis factor-alpha [TNF-α], interleukin-6 [IL-6]) and a decrease in protective adiponectin. These signals activate intracellular inflammatory signaling pathways (e.g., nuclear factor kappa B [NF-κB]) within the vascular wall (Tier 2), leading to amplified cytokine production. This creates a chemotactic gradient that recruits and polarizes immune cells (e.g., macrophages to the pro-inflammatory M1 phenotype) to the perivascular space (Tier 3). The concerted action of this cascade results in a sustained inflammatory attack, causing endothelial cell (EC) dysfunction and inducing osteogenic transdifferentiation of vascular smooth muscle cells (VSMCs)—a key event in calcification initiation. (Created with BioRender.com).

Within the pathogenic network driven by aged EAT, chronic inflammation constitutes the most direct and immediate assault front. Dysfunctional EAT does not release inflammatory signals in a disorganized manner; rather, it mounts a precise attack on the vascular wall through an orchestrated cascade involving adipokine dysregulation, activation of inflammatory networks, and subsequent immune cell recruitment (7, 24). This complete “assault program” and its destructive consequences on the vascular wall are integrated and summarized in **Figure 2**.

#### 4.1.1 Imbalance of the adipokine secretome: a reversal of the pro-/anti-inflammatory ratio

The primary molecular event through which aged EAT drives local inflammation is a fundamental and systemic reprogramming of its adipokine secretome (**Table 1**). This remodeling represents a shift from a predominantly protective, homeostatic profile to a pathogenic, assault-oriented one. It is characterized by a significant upregulation of multiple pro-inflammatory adipokines (e.g., TNF-α, IL-6, resistin, visfatin, leptin) alongside a marked reduction in the secretion of key anti-inflammatory and vasoprotective adipokines (e.g., adiponectin, adrenomedullin, omentin) (37, 38). This transition from a “protective” to an “assault” secretory profile is the most direct molecular manifestation of EAT’s functional switch. It constitutes the initiating driver (Tier 1) of the inflammatory cascade, as depicted in **Figure 2**.

**Table 1:** Age-related dysregulation of the adipokine secretome in epicardial adipose tissue.

Adipokine	Main functions	Change in aging EAT	Selected references
Pro-inflammatory adipokines			
TNF-α	Promotes VSMC migration, proliferation, apoptosis; induces inflammatory signaling.	↑	(25, 26)
IL-6	Drives VSMC phenotypic transformation and plaque instability.	↑	(27)
IL-1β	Inhibition of adiponectin secretion; activation of NLRP3 inflammasome during aging.	↑	(6, 28)
IL-8	Lipolysis induction; neutrophil recruitment and activation.	↑	(6, 29)
**Leptin**	Regulates immune cell function; upregulates endothelin-1; promotes VSMC proliferation and migration.	↑	(30)
**Resistin**	Associated with insulin resistance, angiogenesis, thrombosis, and VSMC migration/proliferation.	↑	(17, 31, 32)
**Visfatin**	Promotes cell proliferation; activates monocytes/macrophages; involved in vascular inflammation and remodeling.	↑	(33, 34)
JNK	Insulin resistance; chronic inflammation; macrophage polarization.	↑	(35)
MCP-1	Mediates chemotaxis of inflammatory cells; contributes to endothelial injury and repair.	↑	(36)
Anti-inflammatory and protective adipokines			
**Adiponectin**	Vascular protection; enhances NO production; inhibits mature macrophage function; improves insulin sensitivity.	↓	(37, 38)
**Omentin**	Improves cardiac function; protects endothelial cells; inhibits oxidative damage; reduces insulin resistance.	↓	(39, 40)
**Adrenomedullin**	Protects vascular endothelium; promotes NO production; modulates calcium signaling.	↓	(41, 42)
IL-10.	Macrophage efferocytosis; immune suppression; resolution of inflammation.	↓	(6, 43)

*CAC, coronary artery calcification; EAT, epicardial adipose tissue; IL, interleukin; MCP-1, monocyte chemoattractant protein-1; TNF-α, tumor necrosis factor-alpha; VSMC, vascular smooth muscle cell.*

↑ indicates increased expression/secretion in aged EAT; ↓ indicates decreased expression/secretion. Bold font highlights adipokines emphasized as major contributors in the main text.

This imbalanced secretome establishes the initial chemical foundation and a persistent stimulus for the chronic low-grade inflammatory milieu surrounding the coronary arteries. These aberrantly expressed adipokines act as dysregulated signaling cues, directly targeting vascular endothelial cells (ECs) and smooth muscle cells (VSMCs) through endocrine and paracrine pathways (44). They not only trigger intrinsic pro-inflammatory signaling within these cells (7), but also orchestrate the expression of a broader spectrum of downstream inflammatory factors, thereby paving the way for the amplification of the subsequent inflammatory response.

#### 4.1.2 Activation of vascular calcification signaling pathways by inflammatory factors

The inflammatory state initiated by adipokine imbalance rapidly culminates in a self-reinforcing and highly activated inflammatory network within the dysfunctional EAT. As illustrated in **Figure 2**, clinical studies demonstrate a prevalent elevation of inflammatory factors alongside a significant reduction in the anti-inflammatory adipokine adiponectin in the EAT of elderly patients with coronary artery disease (24). Key pro-inflammatory signaling pathways, notably those involving NF-κB, JNK, TNF-α, IL-1, and IL-6, are aberrantly activated (45). The initiation of this chronic inflammatory state may be linked to the activation of Toll-like receptors (TLRs) by stimuli such as lipopolysaccharide, which in turn promotes the nuclear translocation of critical transcription factors like NF-κB, leading to the systematic upregulation of inflammatory mediators including IL-6, IL-1, and TNF-α (35).

These inflammatory factors released from EAT act in a paracrine manner on the adjacent coronary arteries (5), exerting continuous stimulation on vascular cells. This stimulation not only directly induces phenotypic changes in ECs and VSMCs but also prompts them to release further cytokines, creating a pro-calcific positive feedback loop (46). Multiple studies have delineated specific mechanistic pathways: for instance, TNF-α and IL-1β can potentiate the effect of BMP-9 via the BMPR2-JNK pathway, promoting endothelial-mesenchymal transition (EndMT) in endothelial cells, thereby initiating calcification (47); they can also facilitate this process through alternative pathways such as cAMP and NF-κB 44. Furthermore, IL-6 expression is significantly upregulated in calcified VSMCs and animal models, while its inhibition (e.g., via puerarin-mediated interference with the NLRP3/NF-κB signaling pathway) effectively attenuates calcification (48, 49).

In summary, inflammatory factors released from dysfunctional EAT convert inflammatory “chemical signals” into “biological instructions” that drive the calcification program by activating a series of well-defined signal transduction pathways within the vascular wall. However, translating these instructions into tangible pathological damage requires an efficient “execution force.”.

#### 4.1.3 Immune cell recruitment and polarization: amplifiers of the inflammatory response

Once inflammatory factors convert “chemical signals” into “biological instructions,” a more destructive cellular cascade is triggered. A potent chemotactic gradient, shaped by both the imbalanced adipokines and the highly activated inflammatory network, robustly promotes the directional recruitment and local activation of immune cells. This process amplifies and materializes the inflammatory response. Studies indicate that chemokines secreted by the EAT of elderly coronary artery disease patients can guide the accumulation of macrophages and T cells around the coronary arteries (50).

Under the influence of factors such as TNF-α and MCP-1, these cells—particularly macrophages polarized towards the pro-inflammatory M1 phenotype—become the core effectors of sustained inflammation (51). As active “executioners,” they release even more potent inflammatory mediators, forming a self-reinforcing inflammatory positive feedback loop (52). Acting in a paracrine manner on the coronary artery wall (5), the inflammatory mediators released by this loop cause vascular endothelial injury and persistently stimulate VSMCs, inducing their pathological transdifferentiation, thereby actively initiating CAC (53).

Moreover, this localized inflammatory storm echoes and interacts with systemic inflammatory status. The neutrophil-to-lymphocyte ratio (NLR), a marker of systemic inflammation, is not only an independent risk factor for coronary artery disease but also positively correlates with EAT volume (54). Clinical studies further confirm that NLR independently correlates with the severity of CAC (55), and, in combination with age, provides a better predictive value for CAC risk (56). This macro-level evidence of “local-systemic” inflammatory crosstalk solidifies the indispensable bridging role of immune cells as both executors and amplifiers of the inflammatory response in EAT-mediated calcification.

**Figure 3:**
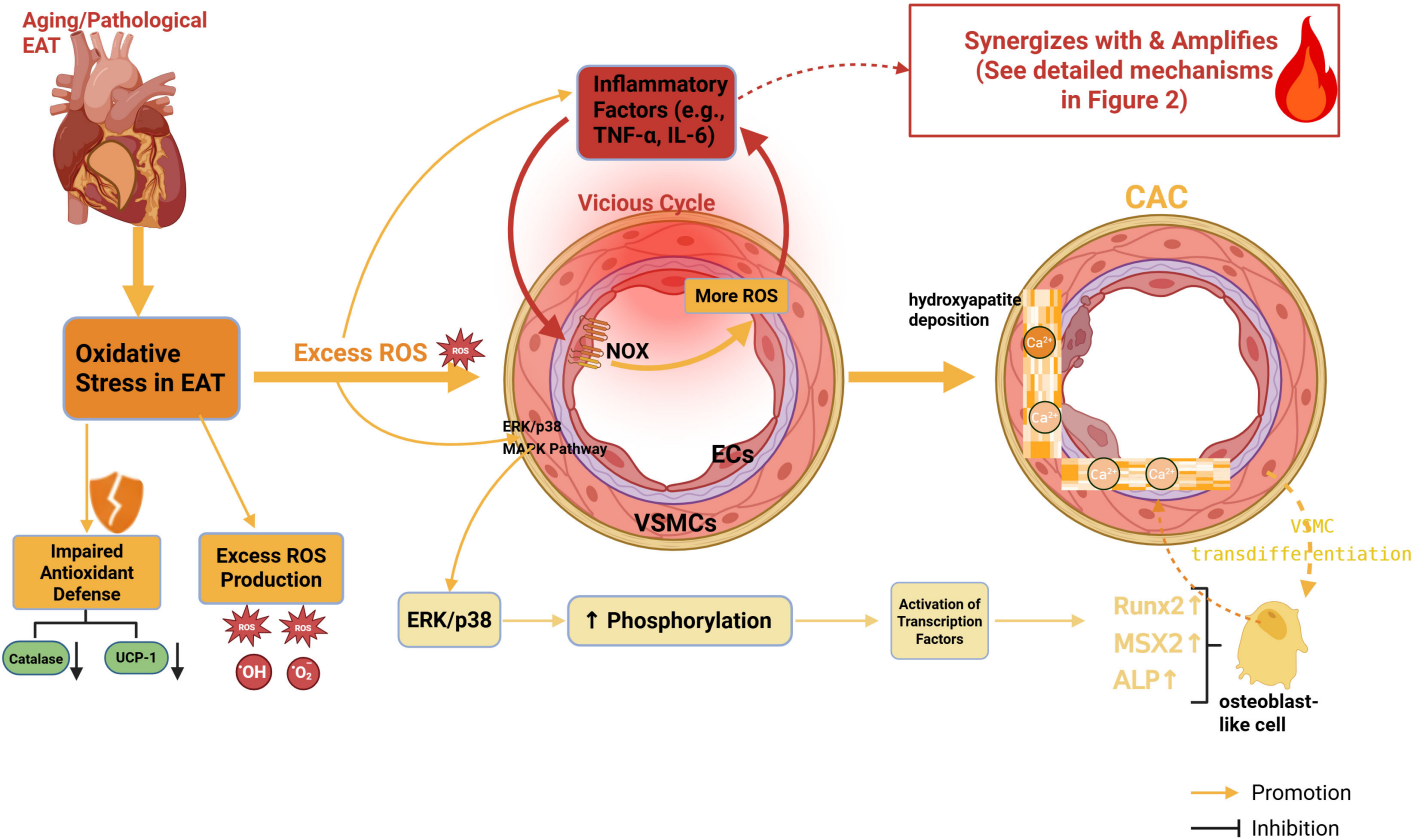
Oxidative stress: a self-amplifying vicious cycle central to the calcification network.This figure highlights the pivotal role of reactive oxygen species (ROS) in creating a self-reinforcing pathogenic axis with inflammation. In aged EAT, excessive ROS production (e.g., via NADPH oxidases [NOX]) coincides with weakened antioxidant defenses. This establishes a vicious cycle: EAT-derived ROS promote inflammation, whose mediators further activate ROS-producing enzymes like NOX in the vascular wall, generating more ROS. This sustained oxidative assault drives calcification through two principal pathways: 1) direct EC injury and 2) induction of osteogenic transdifferentiation in VSMCs, characterized by upregulation of master regulators like Runt-related transcription factor 2 (Runx2). Key pathways such as extracellular signal-regulated kinase/p38 mitogen-activated protein kinase (ERK/p38 MAPK) transduce these oxidative signals. (Created with BioRender.com).

In summary, aged EAT launches a sustained attack on the coronary vascular wall through an orderly, three-tiered inflammatory cascade (**Figure 2**), leading to endothelial dysfunction and driving the osteogenic transdifferentiation of VSMCs. We have thus revealed how EAT acts as both the “igniter” and “accelerant” of the pro-calcific inflammatory fire. However, for this fire to ignite the irreversible calcification program, a critical step that converts chemical signals into sustained cellular damage is required. Notably, the substantial amounts of reactive oxygen species (ROS) generated during this process and inflammatory mediators mutually stimulate each other (as indicated by the connection between **Figure 2** and **3**), tightly coupling inflammation with oxidative stress. This marks a pivotal transition: the local inflammatory attack begins to deeply integrate with systemic redox imbalance. Together, they form a self-reinforcing “core driving axis” within the injury microenvironment (**Figure 1**), propelling the pathological process to a deeper, more consequential stage.

### 4.2 Oxidative stress

As indicated in the preceding section, the inflammatory fire must deeply couple with oxidative stress to form the “core driving axis” of the calcification network (57). In the pathological context of aged EAT, this axis manifests as a state of persistent redox imbalance: both clinical and basic research confirm that dysfunctional EAT is not only a significant source of reactive oxygen species (ROS) but also exhibits a relatively weakened antioxidant defense system (e.g., reduced catalase activity) (58, 59). This imbalance, characterized by excessive production over impaired clearance, is not merely a static background condition. Instead, it engages in a mutual feed-forward relationship with inflammation, establishing a self-perpetuating vicious cycle. **Figure 3** accurately delineates the dynamics of this cycle and elucidates two central pathways through which EAT-derived oxidative stress drives calcification: first, by amplifying vascular damage through crosstalk with inflammatory signaling; and second, by directly programming the osteogenic transdifferentiation of vascular smooth muscle cells.

The following sections will detail the mechanisms by which EAT-derived oxidative stress drives CAC, focusing on two interrelated aspects: excessive ROS production and impaired antioxidant defense.

#### 4.2.1 Excessive production of ROS

Excess ROS originating from dysfunctional EAT drives CAC synergistically through two principal pathways: exacerbating inflammation and directly reprogramming cellular phenotype. First, ROS can potently activate inflammatory signaling pathways (60). Pro-inflammatory factors (e.g., IL-6, TNF-α) released by EAT under oxidative stress stimulate NADPH oxidases in vascular endothelial cells, leading to further ROS generation. This establishes a vicious cycle in which oxidative stress and chronic inflammation mutually reinforce each other (57, 61). Such synergistic damage persistently injures the vascular endothelium, creating a pathological foundation for calcium deposition (62).

Second, ROS can directly target the vascular wall. For instance, ROS such as hydrogen peroxide can induce osteogenic transdifferentiation of VSMCs, characterized by the upregulation of key osteogenic genes like Runx2 and ALPL, thereby directly driving calcification (63–65). Conversely, the application of antioxidants can inhibit this process (66). Research further suggests that NADPH oxidase and its downstream ERK signaling pathway play pivotal roles in this mechanism (67), and their enhanced activity correlates positively with the clinical severity of CAC (68, 69).

Consequently, excessive ROS derived from EAT serves as a central player in advancing coronary calcification, both by amplifying damage signals through crosstalk with inflammation and by directly reprogramming the phenotype of vascular cells.

#### 4.2.2 Weakening of the antioxidant defense system

Concurrent with excessive ROS production, the antioxidant defense capacity of EAT also declines during aging, further exacerbating the redox imbalance. The expression of the key antioxidant enzyme catalase is significantly lower in EAT compared to other adipose depots, impairing the tissue’s ability to detoxify H_2_O_2_ (58). Additionally, the expression and function of uncoupling protein 1 (UCP-1), which exerts antioxidant effects by mitigating mitochondrial oxidative stress, are compromised in aged EAT, leading to a diminution of its protective role (70). This systemic weakening of antioxidant defenses disrupts redox homeostasis, rendering the self-perpetuating vicious cycle depicted in **Figure 3** more likely to initiate and persist.

Therefore, EAT-derived oxidative stress is far from a static “state of injury.” It constitutes a dynamic, self-amplifying vicious cycle (**Figure 3**) that, in a symbiotic relationship with inflammation, forms the core driving axis of the network model (**Figure 1**). This persistent redox pressure acts as a cumulative “oxidative load” on vascular cells, fundamentally destabilizing cellular homeostasis and forcing a critical fate decision: whether to activate autophagy for repair or to succumb to apoptosis. Thus, oxidative stress serves as a key instigator that triggers the dysregulation of the cellular fate decision program—namely, the imbalance between autophagy and apoptosis.

### 4.3 Autophagy and apoptosis

**Figure 4:**
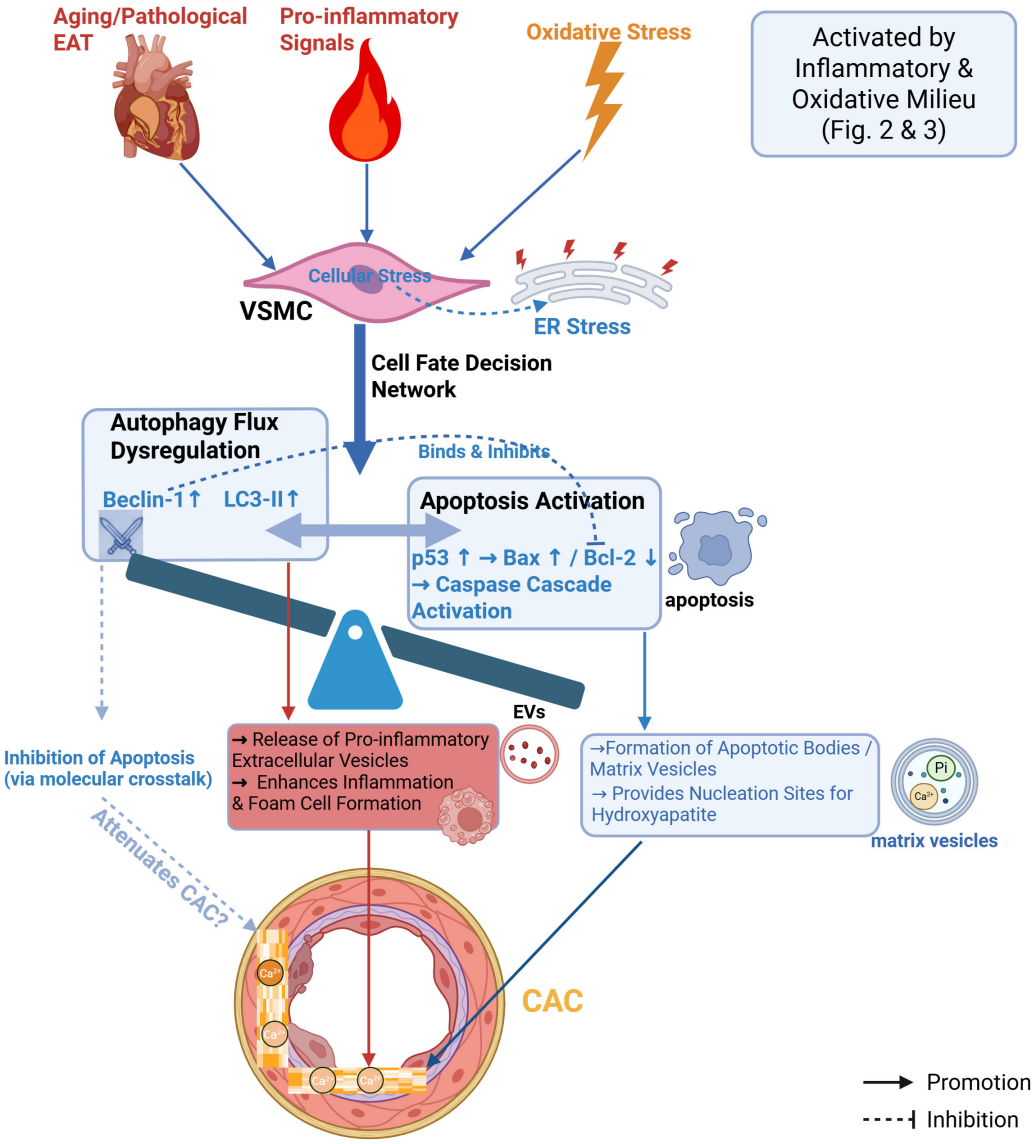
Dysregulation of autophagy and apoptosis in vascular cell fate decisions.This diagram illustrates how vascular cell fate decision-making networks are imbalanced and drive CAC in the inflammatory and oxidative stress microenvironment. Autophagy is enhanced in lesion EAT, but its role is complex: on the one hand, dysregulated autophagy may exacerbate calcification by releasing inflammation-promoting autophagy vesicles (a type of extracellular vesicle); On the other hand, it may also exert a potentially protective effect by removing damaged organelles. However, under the continuous pathological attack, disruption of autophagy homeostasis creates conditions for apoptosis. Factors released by lesion EAT (e.g., TNF-α, ROS) induce mitochondria-dependent apoptosis by activating pathways such as p53-Bax/Bcl-2. Apoptotic bodies (stromal vesicles) released by apoptotic VSMCs provide a key nucleation site for hydroxyapatite crystallization, while intracellular phosphate accumulation activates procalcification signaling, thereby directly translating upstream damage into irreversible calcification outcomes. The crosstalk between autophagy and apoptosis further regulates this cellular fate decision-making process. (Created in BioRender.com). EVs, extracellular vesicles.

At the “fateful crossroads” created by oxidative stress, the life-or-death decision of vascular cells hinges on the balance between their autophagy and apoptosis programs. Under the influence of the pathological microenvironment generated by aged EAT, this decision-making system becomes profoundly dysregulated. Autophagy and apoptosis cease to be mere homeostatic regulators; instead, they collectively evolve into a dysfunctional “cell fate decision network” that actively propels CAC. This complex mechanistic interplay, metaphorically represented as a tilted scale, is summarized in **Figure 4**.

#### 4.3.1 The complex dual role of autophagy in CAC

Autophagy is a core cellular mechanism for maintaining homeostasis, yet its dysregulation is closely associated with the development and progression of cardiovascular diseases (71). In aged and dysfunctional EAT, a state of autophagic flux dysregulation is commonly observed, characterized by elevated levels of autophagy-related proteins (e.g., Beclin-1 and LC3B-II) and gene expression (72, 73). This heightened autophagic activity may be linked to increased AMP-activated protein kinase (AMPK) signaling within the EAT (73).

However, enhanced autophagy exerts a complex and seemingly paradoxical dual effect on CAC, akin to a double-edged sword.

##### 4.3.1.1 Pro-calcific effects

On one hand, dysregulated autophagy within EAT can exacerbate local inflammation. A study by Zhou et al. found that autophagy imbalance leads to increased secretion of pro-inflammatory factors and decreased protective adiponectin from EAT (74). More critically, autophagic vesicles released during this process, as a type of extracellular vesicle (EV), can promote leukocyte adhesion and foam cell formation, thereby accelerating atherosclerosis and calcification (75, 76).

##### 4.3.1.2 Potential protective effects

On the other hand, in specific experimental models, enhanced autophagy has also been shown to mitigate CAC by clearing damaged organelles, alleviating endoplasmic reticulum stress, and suppressing excessive apoptosis (74, 77). For instance, upregulation of the key autophagy protein Beclin-1 can bind to the apoptosis regulator Bcl-2 and inhibit its pro-apoptotic function (78–81).

In summary, within the complex microenvironment of dysfunctional EAT, the net effect of autophagic activation is likely skewed toward harm (as visually suggested by the left side of the scale in **Figure 4**). Its potential cytoprotective benefits are likely overshadowed or even reversed by the sustained inflammatory assault emanating from the aged EAT. More critically, autophagic dysregulation—whether excessive or insufficient—directly compromises cellular homeostasis, thereby setting the stage for the activation of a more terminal pathway: apoptosis (78). Apoptosis, in turn, emerges as the more direct and unequivocal “executor” driving CAC.

#### 4.3.2 Cell apoptosis: the direct terminal pathway driving CAC

Following the disruption of autophagic homeostasis, cell apoptosis emerges as the more direct and unequivocal “executor” linking EAT-derived injury signals to CAC. Inflammatory factors (e.g., TNF-α), adipokines, and oxidative stress released by aged EAT can trigger the mitochondrial apoptosis pathway in VSMCs by activating classical cascades such as p53-Bax/Bcl-2 (82–84) The right, lowered pan of the scale in **Figure 4** visually represents this dominant pathway. The initiation of apoptosis itself constitutes a central event in calcification: apoptotic bodies (matrix vesicles) released by dying VSMCs provide the most critical initial nucleation sites for hydroxyapatite crystal deposition (82). Concurrently, intracellular phosphate accumulation during apoptosis can further activate downstream pro-calcific signaling (85, 86). Therefore, as indicated by the converging point at the bottom of **Figure 4**, vesicles potentially derived from autophagy and apoptotic bodies predominantly generated by apoptosis collectively drive hydroxyapatite deposition, executing the final step of calcification.

Thus, within the microenvironment orchestrated by dysfunctional EAT, the balance between autophagy and apoptosis is disrupted, giving rise to a cell fate decision network skewed toward a pro-calcific outcome (**Figure 4**). This network effectively translates upstream inflammatory and oxidative damage into an irreversible calcific end-point. However, the story does not end here. The substantial accumulation of apoptotic cells and their released matrix vesicles—akin to the “cellular debris” left on the vascular wall—profoundly alters the biochemical composition and biomechanical properties of the extracellular matrix (ECM). This altered landscape, in turn, provides both the impetus and the substrate for the next pathological phase: an active yet maladaptive “reconstruction process” known as vascular remodeling.

### 4.4 Vascular remodeling

Within the pathogenic network driven by aged EAT, if inflammation and oxidative stress initiate the attack and apoptosis leaves behind cellular ‘debris,’ then vascular remodeling constitutes a crucial “structural reorganization layer.” It engages in a pathological reconstruction of this debris, aiming to erect a permanent “scaffold” conducive to calcium salt deposition. The core of this structural derangement lies in the excessive secretion of proteases from dysfunctional EAT, which leads to the systematic degradation of the vascular wall’s extracellular matrix (ECM)—particularly elastin (87, 88). Elastin degradation facilitates calcification through at least two primary mechanisms:

#### 4.4.1 Providing direct nucleation sites

Degraded ECM fragments exhibit a markedly increased affinity for calcium-phosphate crystals, serving as the initial deposition core for microcalcification 88. (89).

#### 4.4.2 Programmed induction of osteogenic transdifferentiation

Biomechanical and biochemical signals released by ECM degradation products (e.g., elastin fragments) can specifically upregulate key osteogenic factors such as bone morphogenetic protein-2 (BMP-2). This, in turn, activates downstream master transcription factors like Runx2 (also known as CBFA1) and MSX2, thereby programmatically driving the transdifferentiation of VSMCs from a contractile to an osteogenic phenotype (90, 91).

This mechanism is substantiated at multiple research levels: in animal models, knockout of MMP-2/MMP-9 genes concurrently suppresses elastin degradation and CAC (92); clinically, the activity and levels of MMPs in the EAT of elderly patients with coronary artery disease are significantly higher than in non-patients and correlate with the degree of calcification (93).

**Figure 5:**
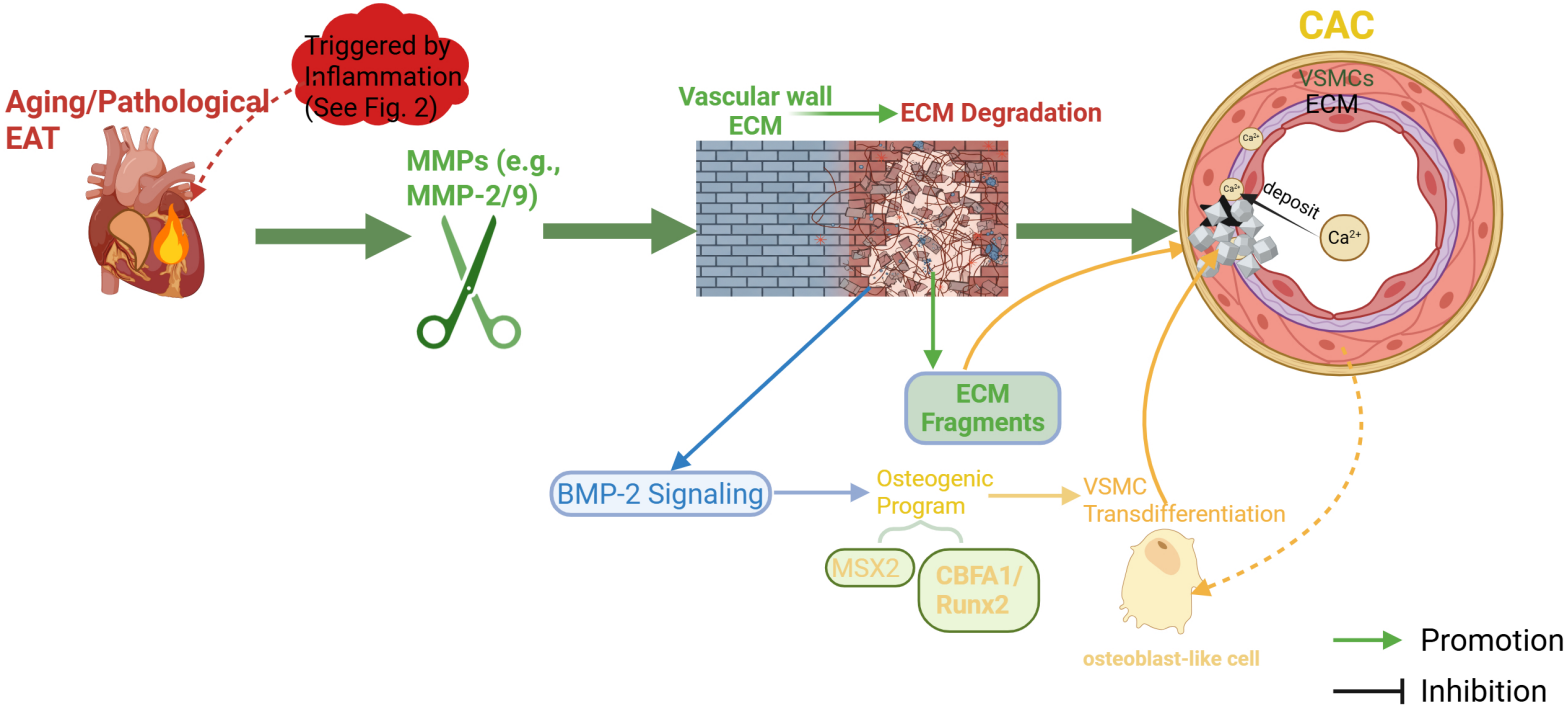
Vascular remodeling: pathological extracellular matrix alterations as a scaffold for calcification.This figure depicts how aged EAT drives structural remodeling of the vascular wall to facilitate CAC. Secretion of matrix-degrading enzymes (e.g., matrix metalloproteinase-2 and -9 [MMP-2/MMP-9]) from EAT leads to proteolytic degradation of the extracellular matrix (ECM), symbolized by the disintegration of an ordered scaffold, with key components like elastin fragmented. These degradation products serve dual roles: they act as direct nucleation sites for calcium-phosphate crystals, and they release bioactive molecules (e.g., bone morphogenetic protein-2 [BMP-2]) that activate pro-osteogenic signaling within VSMCs. This signaling, crucially mediated by the upregulation of Runx2, programs the transdifferentiation of VSMCs into osteoblast-like cells, which actively deposit hydroxyapatite into the disorganized ECM. (Created with BioRender.com).

Consequently, EAT-driven vascular remodeling (**Figure 5**), by deconstructing the organized vascular ECM into a pro-calcific, disorganized milieu, provides the ultimate “landing field” for CAC—both in physical space and through biochemical signaling (94). The engagement of this “structural remodeling layer” signifies a critical transition from reversible cellular signaling disturbances to irreversible tissue-level structural pathology. This orchestrated pathological cascade, from initiation to remodeling, does not occur randomly. It operates as a coordinated network, subject to an even more refined level of global, post-transcriptional regulation—namely, an upstream command network constituted by a spectrum of dysregulated microRNAs (miRNAs) within the EAT.

### 4.5 miRNAs: the upstream regulatory network in diseased EAT

If inflammation, oxidative stress, cell death, and vascular remodeling represent distinct “task forces” executing the attack, what then serves as the “upstream command center” coordinating this multi-tiered assault? The answer lies in a specific spectrum of microRNAs (miRNAs) that become dysregulated within the diseased EAT. These small (~22 nucleotide) non-coding RNA molecules constitute a sophisticated “regulatory network” capable of global integration and precise fine-tuning, thereby orchestrating the entire downstream pathological cascade (95).

#### 4.5.1 Dysregulated expression and functions of key miRNAs

Integrated analyses of EAT from elderly patients with coronary artery disease have identified a distinct miRNA signature, comprising 15 significantly upregulated and 14 downregulated species (96). Among these, miR-135b-3p demonstrates the most pronounced upregulation. This miRNA exerts a dual pathogenic influence: first, it may impair endothelial cell proliferation, migration, and angiogenesis by targeting huntingtin-interacting protein 1 (97);second, it promotes ferroptosis in cardiomyocytes by suppressing glutathione peroxidase 4 (GPX4) expression (98). The ferroptotic process releases a plethora of pro-inflammatory factors, which can perpetuate inflammatory responses, exacerbate endothelial injury and oxidative stress, and drive VSMC transdifferentiation—thereby collectively propelling calcification (99).

These dysregulated miRNAs do not function in isolation but rather form an intricate, coordinated regulatory network. Other altered miRNAs are postulated to participate in this process through distinct pathways. For example, it has been proposed that lipid metabolism disorders may indirectly upregulate miR-455b-3p, which could then exacerbate inflammation and promote calcification by modulating adiponectin (APN) expression, thereby influencing the secretion of adipokines such as KLF4, IL-6, and MCP-1 (97). Furthermore, miR-34a (a member of the miR-34 family) is elevated in the EAT of patients with sudden cardiac death. Experimental studies indicate that it directly promotes VSMC calcification by downregulating the expression of the deacetylase SIRT1 and the AXL receptor tyrosine kinase (100). Collectively, these miRNAs weave a complex signal-integrating network that ensures aging-related signals are accurately interpreted and translated into a coordinated, multi-layered pathological attack.

#### 4.5.2 The miRNA network as an upstream master regulator

**Figure 6:**
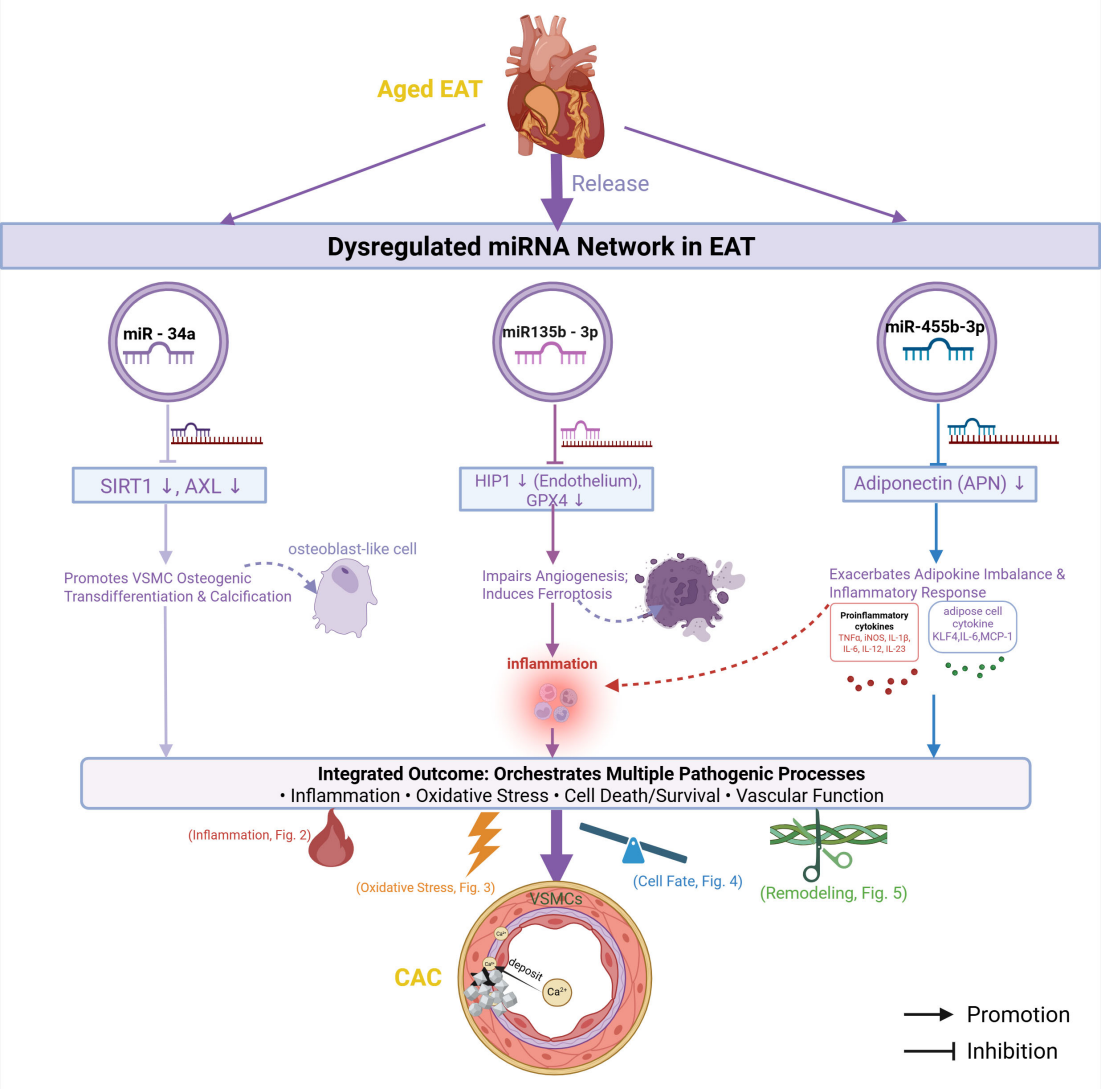
The dysregulated microRNA network in aged EAT as a master regulatory tier. This schematic positions the specific spectrum of microRNAs (miRNAs) altered in aged EAT as an upstream regulatory hub that orchestrates the entire downstream pathogenic network. Key upregulated miRNAs (e.g., miR-135b-3p, miR-34a) within this hub collectively and coordinately exert overarching control over the four core pathological processes detailed in **Figure 2**-**5**: Inflammation, Oxidative Stress, Cell Fate Decisions (Autophagy/Apoptosis), and Vascular Remodeling. For instance, evidence indicates miR-135b-3p exacerbates oxidative stress and inflammation by targeting glutathione peroxidase 4 (GPX4), while miR-34a promotes VSMC calcification by repressing sirtuin 1 (SIRT1). By integrating and amplifying signals across these interconnected layers, the aged EAT-derived miRNA network ensures the potent and synchronized propagation of pro-calcific signals toward CAC. (Created with BioRender.com).

In summary, the specific alterations in the miRNA expression profile within aged EAT act as the “master upstream switch” that activates the entire mechanistic network driving CAC. As summarized in **Figure 6**, this network functions as a central coordinator. It receives and amplifies senescence-associated signals, then precisely orchestrates the downstream “forces”—inflammation, oxidative stress, cell death, and vascular remodeling—launching a concerted pathological assault on the coronary artery wall. Thus, the complete pathogenic circuit—initiated and integrated by the miRNA network (**Figure 6**), then executed through the inflammation-oxidative stress axis (**Figures 2** , **3**), the cell fate network (**Figure 4**), and the structural remodeling layer (**Figure 5**)—is presented comprehensively within our integrated network model (**Table 2**).

**Table 2:** The multi-tiered network orchestrated by aged epicardial adipose tissue in driving coronary artery calcification.

Network tier and role	Core pathological processes	Key effectors and mediators	Direct pro-calcific effect	Inter-tier crosstalk
Upstream Regulatory Tier *(Master Regulator)*	Global post-transcriptional dysregulation of miRNA expression profile.	miR-135b-3p, miR-34a, miR-455b-3p, and others.	Indirect yet pivotal: amplifies pro-calcific signals by fine-tuning all downstream tiers.	**→ All downstream tiers**: Targets transcripts for inflammatory factors, redox enzymes, apoptosis regulators, and matrix proteases.
Core Driving Axis *(Microenvironment Engine)*	1. **Chronic Inflammation:** Adipokine imbalance, cytokine storm, immune cell infiltration. 2. **Oxidative Stress**: Excessive ROS generation coupled with impaired antioxidant defense.	TNF-α, IL-6, Leptin (↑), Adiponectin (↓); M1 macrophages. NADPH oxidases, H_2_O_2_; Catalase (↓), UCP-1 (↓).	1. Establishes a perpetually damaging milieu, inducing endothelial injury and VSMC osteogenic transdifferentiation. 2. Directly activates osteogenic programs (e.g., via Runx2) and exacerbates inflammatory damage.	**↔ Internal Crosstalk:** Inflammation and oxidative stress form a **self-reinforcing positive feedback loop**, constituting the foundational microenvironment. → **Downstream Trigger**: Collectively initiates dysfunction in cellular fate decisions.
Cellular Fate Decision Tier *(Execution Switch)*	1. **Autophagy Dysregulation:** Functional perturbation and vesicle release. 2. **Accelerated Apoptosis**: Programmed cell death activation.	Beclin-1, LC3-II; p53, Bax/Bcl-2 ratio, Caspases; Apoptotic bodies.	1. (Autophagy) Releases pro-inflammatory extracellular vesicles, indirectly promoting calcification. 2. (Apoptosis) Directly provides nucleation sites for hydroxyapatite crystallization via apoptotic bodies—a terminal execution step.	**← Upstream**: Induced by cytokines/ROS. **→ Provides substrate**: Apoptotic debris initiates matrix remodeling. **Internal dialogue**: Molecular interplay (e.g., Beclin-1/Bcl-2).
Structural Remodeling Tier *(Matrix Scaffolding)*	**Vascular Remodeling:** Pathological degradation and rebuilding of the ECM, particularly elastin.	MMP-2, MMP-9; Elastin fragments; BMP-2, Runx2.	1. Degradation products act as direct nucleating cores for calcium-phosphate deposition. 2. Activates osteogenic signaling, programmatically engineering a pro-calcific matrix environment.	**← Upstream inputs**: Modulated by miRNAs, cytokines. Influenced by cell fate debris. **→ Feedback**: Altered ECM cues influence VSMC phenotype.

•CAC, coronary artery calcification; ECM, extracellular matrix; EAT, epicardial adipose tissue; ROS, reactive oxygen species; VSMC, vascular smooth musc cell.

•Arrows indicate direction of primary influence or crosstalk: → (promotes/induces), ← (is induced by), ↔ (bidirectional interaction).

•↑ indicates increased expression/activity; ↓ indicates decreased expression/activity.

Boldface type in the first column denotes the primary hierarchical tiers and their functional roles within the pathological network orchestrated by aged epicardial adipose tissue. These tiers represent a sequential cascade from upstream molecular regulation (Upstream Regulatory Tier) to final tissue mineralization (Structural Remodeling).

## 5 Conclusion and future perspectives

### 5.1 Core conclusion: the proposal of a new paradigm

In summary, this review consolidates evidence into a coherent paradigm: age-related metabolic-endocrine dysfunction of epicardial adipose tissue (EAT) is not merely a bystander but a central orchestrator of coronary artery calcification (CAC). We propose that the aged EAT governs a multi-tiered metabolic-endocrine network in which a self-reinforcing metabolic-inflammatory axis—fueled by mitochondrial dysfunction, altered adipokine secretion, and oxidative stress—serves as the sustained driving force. This core axis, in turn, propagates signals that dysregulate cellular fate decisions (autophagy/apoptosis) and drive pathological vascular remodeling of the extracellular matrix. Superimposed upon and integrating these layers, a spectrum of dysregulated microRNAs acts as a master regulatory hub, coordinating metabolic, inflammatory, and oxidative signals to amplify the entire network. This interactive network model provides a more holistic understanding of how systemic aging is focally translated into vascular calcification, moving beyond linear pathways to a systems-level perspective. Consequently, therapeutic strategies aimed at reprogramming the aged EAT at its metabolic core—rather than merely targeting its downstream inflammatory products—hold substantial potential for preventing or retarding the progression of CAC in the elderly.

### 5.2 Future perspectives: from network deconstruction to metabolic reprogramming

Translating this metabolic-endocrine network paradigm into clinical impact requires focused efforts on two complementary fronts.

First, deconstructing network complexity. Employing spatial transcriptomics and single-cell technologies is crucial to map the cellular and metabolic heterogeneity within aged EAT and its dynamic communication with the vascular wall. Such approaches will identify the most vulnerable, disease-specific network nodes—whether specific adipocyte subpopulations, immune cell phenotypes, or key metabolic sensors—that can serve as precise intervention targets.

Second, engineering metabolic network interventions. The recognition of EAT as a metabolic disease hub opens a distinct therapeutic frontier: metabolic reprogramming of the dysfunctional EAT itself. Beyond conventional anti-inflammatory or anti-calcific agents, next-generation strategies could aim to correct the root metabolic derangements. These include enhancing mitochondrial bioenergetics and restoring UCP-1 function to improve local energy homeostasis and reduce oxidative stress; modulating lipid turnover via selective PPARγ modulation to restore a healthier balance between lipid storage and release; activating cellular metabolic sensors such as AMPK within EAT adipocytes to re-establish redox equilibrium and a protective secretory profile; and targeting the dysregulated miRNA network using antisense oligonucleotides or mimic technologies to restore normal post-transcriptional regulation. The integration of such metabolic re-tuning with targeted anti-inflammatory or anti-osteogenic agents could yield synergistic effects, attacking the pathological network at multiple interdependent levels.
